# Antimicrobial proteins: intestinal guards to protect against liver disease

**DOI:** 10.1007/s00535-018-1521-8

**Published:** 2018-11-03

**Authors:** Tim Hendrikx, Bernd Schnabl

**Affiliations:** 1 Department of Medicine, University of California San Diego, La Jolla, CA USA; 2 Department of Medicine, VA San Diego Healthcare System, San Diego, CA USA

**Keywords:** Dysbiosis, Innate immune system, Bacterial translocation, Microbiome

## Abstract

Alterations of gut microbes play a role in the pathogenesis and progression of many disorders including liver and gastrointestinal diseases. Both qualitative and quantitative changes in gut microbiota have been associated with liver disease. Intestinal dysbiosis can disrupt the integrity of the intestinal barrier leading to pathological bacterial translocation and the initiation of an inflammatory response in the liver. In order to sustain symbiosis and protect from pathological bacterial translocation, antimicrobial proteins (AMPs) such as a-defensins and C-type lectins are expressed in the gastrointestinal tract. In this review, we provide an overview of the role of AMPs in different chronic liver disease such as alcoholic steatohepatitis, non-alcoholic fatty liver disease, and cirrhosis. In addition, potential approaches to modulate the function of AMPs and prevent bacterial translocation are discussed.

## Introduction

The gastrointestinal tract is the largest surface area in the body and is home to a vast consortium of symbiotic bacteria that play an important role in human health and disease. Microbiota are involved in basic human biological processes, including food digestion, modulation of immune responses, regulation of epithelial development and generation of a variety of products as a result of microbial metabolic activities. These products together with host–bacteria interactions influence both normal physiology and disease susceptibility. A disruption of the symbiosis between microbiota and host is known as dysbiosis and is described in multiple chronic diseases such as obesity [1], malnutrition [2], neurological disorders [3], inflammatory bowel disease [4], diabetes mellitus [5], metabolic syndrome, atherosclerosis [6], cancer [7] and liver disease [8–10].

Nutrition, other environmental and genetic factors can independently cause changes in the gut microbiota composition, which can present as qualitative changes such as increased proportions of harmful bacteria and reduced levels of beneficial bacteria, and also quantitative changes in the total amount of bacteria (intestinal bacterial overgrowth) [10, 11]. Intestinal bacterial overgrowth can affect both the luminal compartment and mucosa-associated bacteria [12]. As a result of dysbiosis, intestinal epithelial integrity is lost, mucus-associated defense is weakened and the intestine becomes more permeable. Hence, viable bacteria or microbial products are able to migrate from the intestines to mesenteric lymph nodes or other extra-gastrointestinal organs via the bloodstream, causing disease [13]. At distant sites, these bacterial products can be recognized via toll-like receptors (TLRs). Specific TLRs recognize pathogen-associated molecular patterns associated with bacteria. TLR-2 may be activated by various membrane components of Gram-positive bacteria. TLR-4 recognizes the lipid A portion of lipopolysaccharide (LPS). TLR-5 may be activated by flagellin, and bacterial DNA activates TLR-9. Activation of TLRs on macrophages leads to a variety of inflammatory cascades that in turn cause inflammation and may represent the driving force behind disease progression [14]. In addition, upon liver injury, hepatic stellate cells undergo an activation process in which they express TLR4. Therefore, LPS and other TLR ligands may enhance fibrogenic responses via direct stellate cell activation. Lastly, as also hepatocytes express TLR-2 and TLR-4, bacterial recognition by TLRs on hepatocytes may account for cell death occurring during liver injury [15].

Besides the tightly interconnected intestinal epithelial lining, the physical barrier to separate microbiota from intestinal surface is formed by a mucus layer. This mucus is secreted by goblet cells and largely consists of mucin glycoprotein sheets. In the colon, where mucus consists of two layers, the inner mucus layer is devoid of bacteria whereas the outer is colonized [16]. Likewise, in the small intestine bacteria are kept on distance from the epithelial wall [17]. In order to sustain the mucosal barrier and protect the host against enteric pathogens, a range of host antimicrobial factors are produced in the intestinal epithelium [18]. These intestinal antimicrobial proteins (AMPs) mediate killing of bacteria by attacking the basic cell wall structures through enzymatic and non-enzymatic mechanisms. Interestingly, dysfunctional antimicrobial defense has been described in different chronic liver diseases [19–21].

**Fig. 1:**
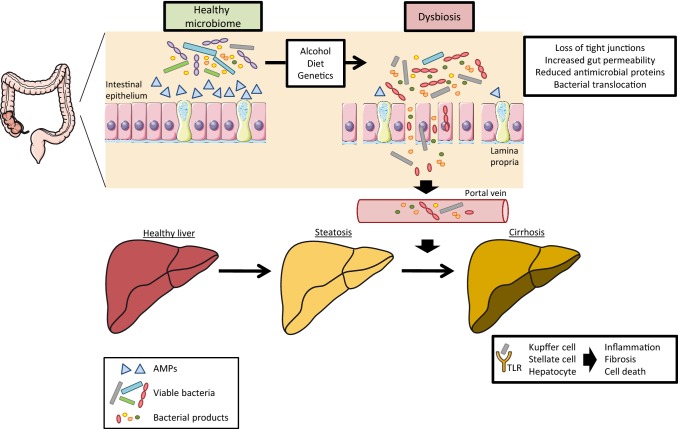
Steatosis (fatty liver) due to obesity (diet), alcohol consumption, other environmental or genetic factors is associated with dysbiosis, loss of intestinal tight junctions, reduced intestinal epithelial integrity, increased permeability and lower expression of antimicrobial proteins (AMPs). This allows bacterial products such as LPS (via the paracellular route) or viable bacteria (via not further detailed mechanisms) to translocate into the bloodstream and mesenteric lymph nodes. Via the portal vein, bacteria and their products reach the liver, where they promote progression to more severe stages of liver disease via recognition by Toll-like receptors (TLRs) on Kupffer cells, hepatic stellate cells and hepatocytes, leading to inflammation, fibrosis and cell death (Figure made using Servier Medical Art; http://smart.servier.com)

In this review, we summarize evidence supporting the contribution of bacterial translocation and the role of antimicrobial proteins in the development and progression of different chronic liver diseases such as alcoholic and non-alcoholic fatty liver disease (NAFLD), and cirrhosis (Fig. 1). Moreover, potential approaches to modulate the function of antimicrobial proteins to prevent bacterial translocation are discussed.

## Antimicrobial proteins

The surface of the mammalian intestine continuously encounters bacteria, fungi, viruses and parasites that could act as pathogens. In order to cope with these microbial challenges, a diverse collection of AMPs are produced in the intestinal epithelium and Paneth cells which rapidly kill or inactivate microorganisms. These AMPs consist of different protein families, which include defensins, cathelicidins, C-type lectins (such as the regenerating islet-derived protein (REG) family), ribonucleases (RNases, such as angiogenin 4) and S100 proteins (such as calprotectin (also known as S100A8–S100A9) and psoriasin (also known as S100A7)) [22]. Although most AMPs attack bacterial cell wall components, different AMP families use distinct molecular mechanisms to kill microorganisms [18]. This might explain that microbial resistance to multiple AMPs is rare.

The expression, secretion and activity of AMPs are under tight control of different factors. First, studies of germ-free mice have revealed that some intestinal AMPs require bacterial signals for their expression, whereas others are expressed independently of the microbiota [18]. For example, the expression of regenerating island-derived protein 3 gamma (REG3γ) is essentially absent in germ-free mice and is upregulated on colonization with a conventional microbiota [23]. Besides bacterial signals, host immune function controls the expression of these bacterially regulated AMPs. Studies in mice have shown that bacterial recognition by TLRs on intestinal epithelial cells is required for and upregulates the expression of REG3γ and REG3β [24, 25]. In addition, intestinal epithelial cell expression of REG3γ also requires interleukin 22 (IL-22), a cytokine mainly expressed by RORγt^+^ type 3 innate lymphoid cells (ILC3s) in the gut [26]. The production of this cytokine by ILC3s is enhanced by activation of the aryl hydrocarbon receptor (AhR) via specific bacterially derived molecules [27–29], indicating that bacteria might enhance the immune response to protect the host from harmful pathogens. Taken together, the expression and function of AMPs are the result of a symbiotic interplay between host and commensals, in which interruptions might cause disease. Here, we further describe the role of AMPs, more specifically C-type lectins, in different chronic liver diseases.

## Alcoholic liver disease

Alcoholic liver disease affects several million people worldwide and can progress from hepatic fat accumulation (steatosis) and alcoholic steatohepatitis to cirrhosis and hepatocellular carcinoma [30]. Based on current understanding, multiple pathogenic factors are involved in the development of alcoholic liver disease [31, 32]. Both clinical and experimental evidence show that alcohol abuse is associated with dysbiosis. Transfer of the dysbiotic intestinal microbiota from alcoholic hepatitis patients to germ-free and conventionalized mice demonstrated that alcohol-associated dysbiosis contributes to the development of alcoholic liver disease [33]. In addition, gut barrier dysfunction and increased intestinal permeability have been implicated in alcohol-induced liver injury [34]. Mechanistically, acetaldehyde, which is a product of ethanol metabolism, and the generation of reactive oxygen species through cytochrome P450 2E1 induction might contribute to tight junction disruption leading to increased permeability during alcohol consumption [35]. In addition, intestinal inflammation is an important mediator of intestinal barrier dysfunction during alcohol consumption [11].

### Bacterial translocation in alcoholic liver disease

In line with increased intestinal permeability, translocation of bacteria has been implicated in the development of alcoholic liver disease. Experimental induction of bacterial overgrowth in the small intestine alone is sufficient to result in bacterial translocation and subsequent liver disease [36]. Inversely, administration of non-absorbable antibiotics prevented bacterial overgrowth, reduced pathological bacterial translocation and ameliorated ethanol-induced steatohepatitis in rodents [37]. Moreover, plasma levels of gut-derived microbial products such as LPS and peptidoglycan are increased during alcohol administration [38, 39]. Recently, our group demonstrated that besides bacteria, also fungi contribute to alcohol-related liver disease. Increased fungal growth and translocation of fungal products to the liver activate inflammatory immune responses in the liver of ethanol-fed mice. This process is mediated via β-glucan recognition by CLEC7A on Kupffer cells. Moreover, relatively to healthy people, alcohol-dependent patients showed altered fungal signature and increased exposure and immune reactivity to fungal products in the blood, indicating fungal translocation [40]. Taken together, these data support that during alcoholic liver disease, epithelial damage in the intestine leads to pathological translocation of microbial products, thereby causing liver damage.

### Antimicrobial defense during alcoholic liver disease

We previously described that chronic alcohol consumption suppresses REG3γ and REG3β mRNA and protein levels in murine small intestine [19, 21], and duodenal REG3γ in patients with alcohol use disorder [21]. In mice, the lowest levels of *Reg3γ* and *Reg3β* were observed in the proximal small intestine, where the bacterial overgrowth was most pronounced and luminal alcohol concentrations are highest [21]. Decreased REG3γ can be restored using prebiotics, which are associated with suppression of intestinal bacterial overgrowth. We have demonstrated that ethanol-fed *Reg3γ*^−/−^ and *Reg3β*^−/−^ mice have increased susceptibility to ethanol-induced liver disease, in association with increased mucosa-associated bacteria and more translocation of bacteria to the liver. In line, intestine-specific overexpression of *Reg3γ* protects mice against ethanol-induced liver disease by maintaining an inner mucus layer devoid of bacteria and reducing bacterial translocation [12]. How a reduced number of mucosa-associated bacteria results in lower bacterial translocation is not known. Further, mice deficient for mucin-2 production that were protected against alcohol-induced liver lesions showed increased defensin production as well as that of *Reg3β* and *Reg3γ* [19]. These data indicate that antimicrobial defense plays an important role in preventing bacterial translocation and protect against alcoholic liver disease development. Other antimicrobial molecules do not seem to be suppressed by chronic ethanol treatment [21], although a global analysis using transcriptomic or proteomic approaches should be done in future studies.

Recent findings from our laboratory suggest that *Reg3γ* and *Reg3β* suppression during alcoholic liver disease is an indirect effect of alcohol consumption. Using chronic–binge ethanol-fed mice as a model of alcoholic steatohepatitis [41], we found that dysbiosis upon ethanol consumption is associated with altered tryptophan metabolism by bacteria [42, unpublished data]. Ethanol feeding resulted in lower levels of indole-3-acetic acid, a ligand for the AhR [29, ], and reduced production of IL-22 by intestinal lamina propria ILC3. Importantly, AhR-dependent production of IL-22 regulates REG3γ and REG3β expression. Administration of non-absorbable antibiotics to ethanol-fed mice restored IL-22 production, indicating the influence of microbiota on regulating IL-22 expression [42, unpublished data]. Taken together, these data suggest that alcohol consumption changes microbial composition, thereby affecting the bacterial metabolome which alters host immunity and allows bacterial translocation.

Nevertheless, the exact mechanism of how chronic ethanol administration results in changes of the luminal intestinal microbiota composition is not fully elucidated. Undoubtedly, chronic alcohol consumption affects multiple factors in the host and more mechanistic studies are needed to fully understand how changes in the gut microbiome impact liver function during alcoholic liver disease and vice versa.

## Non-alcoholic fatty liver disease

The prevalence of NAFLD is increasing worldwide and is considered to be a hepatic manifestation of the metabolic syndrome. Due to its strong association with obesity and type 2 diabetes, the pathogenesis of NAFLD and its progression to more complicated conditions have been widely accepted to be the result of multiple factors including intestinal dysbiosis [43, 44]. Similar to patients with alcoholic fatty liver disease, studies show that a shift in the gut microbiota composition correlates closely with the prevalence and progression of NAFLD. In patients with NAFLD, a decrease of some selected members of Firmicutes has been observed and obese patients with non-alcoholic steatohepatitis (NASH) had reduced Bacteroidetes compared with healthy controls [45]. Severity of NAFLD is associated with gut dysbiosis and microbial metabolome [46, 47]. These studies indicate that an alteration in the composition of the gut microbiota is closely associated with the development of NAFLD.

Different microbiota-dependent mechanisms have been suggested to contribute to NAFLD pathogenesis and progression. Ethanol-producing bacteria were proposed to be more abundant in NASH patients [48]. Further, dysbiosis may result in production and translocation of LPS and other inflammatory factors, changes in bile acid metabolism, and increased gut permeability in a subset of NAFLD patients [49]. This facilitates translocation of bacterial products into the portal circulation and activation of inflammatory processes.

### Bacterial translocation in NAFLD

Studies in rodent models have shown correlations between hepatic inflammation and dysfunction of the intestinal mucosal barrier, which suggest that intestinal mucosal barrier malfunction and bacterial translocation influence the pathogenesis of NAFLD and NASH. Indeed, it has been shown that tight junction disruption in mice and NAFLD patients increases intestinal permeability and bacterial translocation to the liver through the bloodstream [50–52]. Nevertheless, data suggest that only a portion of NAFLD patients have increased intestinal permeability. It was reported that serum endotoxin levels were increased in only 42.1% (8/19) patients with NASH and a meta-analysis found that only 39.1% of patients with NAFLD (*n* = 128) had increased intestinal permeability [53]. Therefore, gut barrier dysfunction with subsequent translocation of microbial products might make only a small contribution to development or progression of fatty liver disease, or only in a subset of patients.

### Antimicrobial defense during NAFLD

Similar to chronic ethanol consumption, animal models of diet-induced obesity indicate a downregulation of intestinal *Reg3γ* [54]. Recently, we explored the role of Reg3 lectins in the development of NASH. To induce NASH, mice deficient for REG3β or REG3γ were fed a Western-style fast-food diet (rich in saturated fat, cholesterol and fructose) for 20 weeks. Loss of REG3β or REG3γ did not cause more severe liver disease than in their WT littermates, despite elevated endotoxemia in *Reg3γ*-deficient mice. In addition, intestinal overexpression of REG3γ did not protect mice against NASH development [55]. Overall, these results indicate that loss of REG3β or REG3γ is insufficient to aggravate diet-induced obesity and NAFLD.

Vitamin D insufficiency, which has been associated with metabolic syndrome and NAFLD, has been found to be associated with loss of Paneth cell defensins, which may consequently lead to intestinal dysbiosis and endotoxemia. Moreover, oral administration of human alpha-defensin 5 rebalanced gut microbiota and resolved hepatic steatosis in mice [56]. Further, cathelicidin, another antimicrobial peptide, suppresses lipid accumulation and hepatic steatosis via the inhibition of the CD36 receptor [57]. Recently, the expression of cathelicidin-related antimicrobial peptide (*Cramp*), the only member of cathelicidin antimicrobial peptide family in mice, was found to be decreased by alcohol exposure to mice [58]. More studies investigating the function of different antimicrobial proteins during fatty liver disease development and progression are needed. Nevertheless, current data support the notion that bacterial translocation might not be as important during NAFLD as observed in patients with alcohol use disorder and alcohol-induced liver disease.

## Cirrhosis

For most chronic liver diseases, cirrhosis is the common end-stage histologic distortion, characterized by the presence of regenerative nodules that causes portal hypertension. This in turn induces bacterial overgrowth by altering intestinal motility [59]. The gut–liver axis is well studied during cirrhosis and complications such as hepatic encephalopathy, spontaneous bacterial peritonitis and variceal bleeding are the result of pathological translocation of bacteria or their products into the blood of cirrhotic patients [60–63]. Evidence from both animal and patient studies indicates a loss of epithelial tight junctions during cirrhosis. Patients or mouse models of liver cirrhosis showed reduced intestinal expression of zonula occludens-1, occludin and claudin-1 compared to controls, and these changes were more evident in cases of decompensated or more advanced stage of disease [64]. Moreover, intestinal permeability is enhanced via increased production of lipid peroxidation products such as malondialdehyde in the intestine, as described in patients and rats with cirrhosis [65, 66].

### Bacterial translocation in cirrhosis

In patients with cirrhosis, changes in gastrointestinal barrier and dysbiosis increase the rate of bacterial translocation [62]. Indeed, cirrhotic patients have increased levels of LPS and bacterial DNA in the portal circulation compared to healthy controls, with increasing amounts as the liver function worsens [67, 68]. Whereas the rate and degree of translocating bacterial products are higher in early cirrhosis compared to healthy conditions, pathological translocation of viable bacteria occurs in the decompensated stage of the disease. During decompensated cirrhosis, a further increase in intestinal permeability could be triggered by intestinal inflammation and may contribute to enhanced translocation of viable bacteria [69, 70]. Living bacteria appear to translocate via the transcellular route (transcytosis), whereas microbial products migrate via disrupted tight junctions using the paracellular route and further enhance the local and systemic inflammatory response [71]. Positive cultures from mesenteric lymph nodes are found in about 50–60% of rats with CCl_4_-induced cirrhosis and in 30% of cirrhotic patients [72, 73], supporting that pathological bacterial translocation plays an important role in disease progression during cirrhosis.

### Antimicrobial defense during cirrhosis

Besides intestinal immune cell damage, several studies point to deficiencies in the production of intestinal antimicrobial peptides during cirrhosis. Cirrhotic rats with ascites and translocation of viable bacteria to mesenteric lymph nodes produce lower levels of defensins molecules compared with cirrhotic rats without bacterial translocation [20]. The transcription factor farnesoid X receptor (FXR), which is the nuclear receptor for conjugated bile acids, plays a crucial role in preserving intestinal epithelial integrity by increasing antimicrobial peptide production and secretion [74]. Treatment of cirrhotic rats with obeticholic acid, a potent agonist of FXR, significantly reduced bacterial translocation from the intestine to the blood via upregulation of antimicrobial proteins angiogenin-1 and α-5 defensin, as well as tight junction proteins, and reduced liver fibrosis [75]. Further, liver cirrhosis in advanced stages is frequently associated with malnutrition [76], which has deleterious effects on gut mucosal integrity and antimicrobial peptides [77]. Data on the expression of intestinal AMPs in cirrhosis are scarce. Mucosal expression of several different AMPs was not altered in the ileum or colon of patients with cirrhosis as compared with healthy controls [78]. Taken together, experimental data in animal models underline the concept of intestinal antimicrobial deficiency in cirrhosis. Measurements of AMPs in more and larger patient cohorts should be undertaken.

## Modulation of antimicrobial proteins as treatment for liver disease

Since bacterial translocation and reduced expression of certain antimicrobial proteins in the gut are observed in rodents and humans with liver disease, designing a strategy to increase intestinal concentrations of antimicrobial proteins or their production by intestinal epithelial cells might be developed to prevent liver disease.

### Antibiotics

Non-absorbable antibiotics have a beneficial effect on NASH [79] and alcoholic liver disease [37], and are commonly used to treat patients with cirrhosis [80]. Although the use of antibiotics seems to have a beneficial effect by reducing bacterial overgrowth and preventing bacterial translocation, no evidence supports that this is achieved by increased expression of antimicrobial proteins. Nevertheless, recent data from our group indicate that non-absorbable antibiotics can restore IL-22 production by ILC3 s during ethanol diet [42, unpublished data]. Therefore, changes in the microbial metabolome, the composition of microbiota, or host immunity due to antibiotics might affect antimicrobial responses indirectly.

### Probiotics and prebiotics

Probiotics regulate antimicrobial defense. For instance, probiotic *Escherichia coli* Nissle 1917 and a variety of other probiotics such as lactobacilli strongly induced the expression of human beta-defensin-2 in epithelial cell lines [81]. In addition, probiotic lactobacilli strains are not only able to upregulate enterocyte human beta-defensin 2 (hBD-2) production in vitro [82]; some species, such as *Lactobacillus lactis*, have been demonstrated to be resistant to the antimicrobial effects of this defensin [83]. Apart from the induction of AMP, probiotics might affect cytokine-producing innate cells in the mucosa (e.g., IL-22) that can increase the expression of Reg3 lectins. As such, probiotic *Lactobacillus reuteri* was found to produce AhR ligands from tryptophan metabolism, thereby enhancing IL-22 production and mucosal defense [29]. This host–commensal interplay is a prime example of how beneficial bacteria might enhance the immune response to protect the host from pathogens.

Prebiotics are complex short-chain saccharides that cannot be digested by host pancreatic and brush-border enzymes, but can be selectively used and fermented by the commensal microbiota. They stimulate probiotic bacteria such as lactobacilli and bifidobacteria [84]. Interestingly, we found that decreased REG3γ level during ethanol-induced liver disease can be partly restored using prebiotics. Adding fructooligosaccharides to ethanol-fed mice reduced ethanol-induced steatohepatitis and intestinal bacterial overgrowth by partial restoration of REG3γ [21]. Current evidence supporting a beneficial effect of prebiotics for NAFLD and cirrhosis is lacking. More studies and larger clinical trials are needed to support the use of pre- and probiotics in different chronic liver diseases.

## Conclusion

In conclusion, several human and mouse studies have demonstrated that intestinal barrier dysfunction, bacterial translocation and a deficiency in various antimicrobial proteins are implicated in the development of chronic liver disease. We are gaining increased insight into the close relationship between the gut and the liver evoked by dysbiosis. The evaluation of the gut–liver axis and the intervention of the relationships between antimicrobial peptides and bacteria might aid the development of treatment and prevention for liver disease patients. Finally, besides bacteria, the intestinal microbiota also includes eukaryotic viruses [85], bacteriophages [86], and eukaryotic organisms such as fungi [87]. However, studies were mainly focused on the interaction between AMPs and bacteria. Therefore, studies to explore the influence of AMPs on other microbial communities will be interesting. In that way, more insights in the communication between host and microbiome will be made which may provide new strategies for improving health and disease management.

## Abbreviations

AhR: Aryl hydrocarbon receptor
AMPs: Antimicrobial proteins
FXR: Farnesoid X receptor
IL-22: Interleukin 22
ILC3: Innate lymphoid cells type 3
LPS: Lipopolysaccharide
NAFLD: Non-alcoholic fatty liver disease
NASH: Non-alcoholic steatohepatitis
Reg: Regenerating island-derived protein
TLR: Toll-like receptor
